# Mental health professionals dealing with aggression: the experience of Ar-Razi hospital in Salé, Morocco

**DOI:** 10.1192/j.eurpsy.2025.1883

**Published:** 2025-08-26

**Authors:** A. Souidi, G. Hami, S. Belbachir, A. Ouanass

**Affiliations:** 1 Ar-razi Hospital, Salé, Morocco; 2 psychiatry B, Ar-razi hospital, Salé, Morocco

## Abstract

**Introduction:**

Aggression in the psychiatric environment has negative consequences for both patients and staff. Accurate measurement of patient aggression towards staff, and its prevention, remains elusive. The Staff Observation of Aggression Scale-Revised (SOAS-R) is a measure describing the incidence and characteristics of aggressive events. Although not exclusive to staff aggression, our study used this scale to provide a more or less standardized measure for assessing and understanding the trends and potential triggers of staff aggression. This will enable better management of such situations. This in turn will lead to better working conditions and improved quality of care.

**Objectives:**

To describe the incidence of aggressive events against various mental health staff at Ar-Razi Hospital over the course of a year, their characteristics and the factors involved. this study will provide and enable better managment of these situations, furthermore leading to better work conditions and better quality of care.

**Methods:**

- Our study is a cross-sectional study, data collection were by a questionnaire sent online to hospital staff, the questionnaire was bases on the SOAS-R items.
- Descriptive and analytical statistical study wad done by Jamovi 2.3 software.
- Statistical differences were calculated using the chi-2 test, and a p<0.005 value was considered statistically significant.

**Results:**

Our study results(SOAS-R items) concerning the agressions towards staff of our hospital were resumed in image 1 and 2.

Also the prevalence of participating staff by status in our study was: 78% of physical assaults were against nurses. The association between the prevalence of assaults and staff status was statistically significant using the chi-2 test (p=0.0037<0.001).

**Image 1:**

**Figure:**
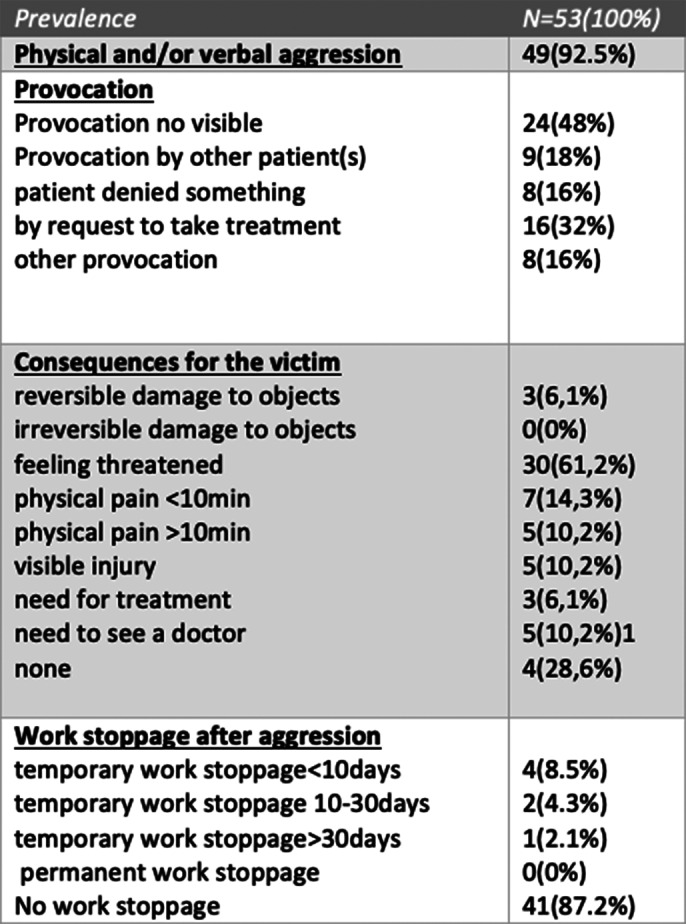

**Image 2:**

**Figure:**
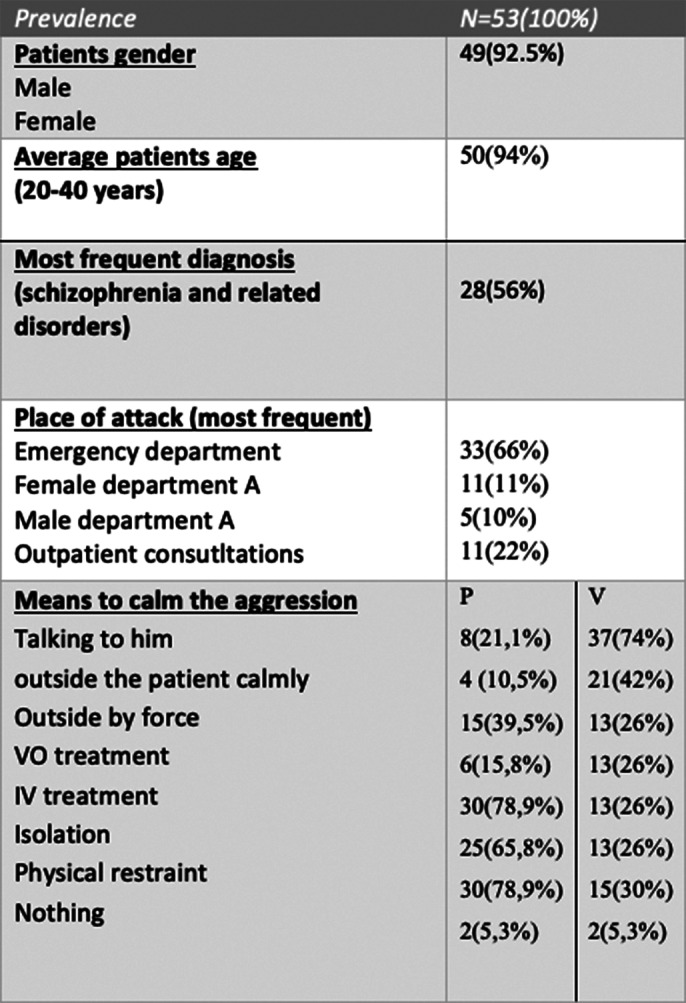

**Conclusions:**

Agression is known to have negative impacts for both staff and patients. Our study suggests that mental health staff in our facility are victims of aggression. Improved methods of measuring aggression and a wider study of different psychiatric institutions will help to prevent aggression and create a healthy work environment, which in turn will help the staff to develop and improve the quality of care for patients.

**Disclosure of Interest:**

None Declared

